# Chloride of Iron in Typhoid Fever

**Published:** 1892-06

**Authors:** 


CHLORIDE OF IRON IN TYPHOID FEVER.—Dr. J. W. Anderson describes in the *British Medical Journal* a mode of treatment for typhoid fever which he has found very effectual. It consists in administering a full dose of the liq. ferri perchloridi fort., namely, 5 minims (for an adult) every hour of the day and night, until a week has elapsed from the complete subsidence of the fever. To enable the patient to take this, the dose is combined with half a drachm of glycerine or 1 drachm of simple syrup, and a few drops of tinct. zingib. fort., and diluted in half a tumblerful of water. If sickness is caused, 5 grains of bismuthi subnit. are given ten minutes before each dose of the medicine until nausea ceases to be produced. In a few days the diarrhœa will be arrested, and thereafter a mild aperient must be given daily as long as the medicine is continued. In a moderately severe case, not brought under this treatment until the end of the first week of fever, it will take, the writer says, ten days to reduce the temperature to normal. If the medicine is not given every hour night and day it will take a little longer; if begun within two or three days of onset of fever, the latter will be gone in about five days.—*Medical Record.*

